# The periprocedural and 30-day outcomes of carotid stenting in patients with carotid artery near-occlusion

**DOI:** 10.1038/s41598-021-01286-3

**Published:** 2021-11-08

**Authors:** Cheng-Hsuan Tsai, Ying-Hsien Chen, Mao-Shin Lin, Ching-Chang Huang, Chi-Sheng Hung, Chih-Fan Yeh, Sheng-Fu Liu, Sung-Chun Tang, Chi-Chao Chao, Hsien-Li Kao

**Affiliations:** 1 Graduate Institute of Clinical Medicine, National Taiwan University College of Medicine, Taipei, Taiwan; 2 Department of Internal Medicine, National Taiwan University Hospital Jinshan Branch, New Taipei City, Taiwan; 3 Division of Cardiology, Department of Internal Medicine and Cardiovascular Center, National Taiwan University Hospital, No. 7, Chung-Shan South Road, Taipei, 100 Taiwan; 4 Department of Internal Medicine, National Taiwan University Hospital, Hsin-Chu Biomedical Park Hospital, Hsin-Chu, Taiwan; 5 Stroke Center and Department of Neurology, National Taiwan University Hospital, Taipei, Taiwan; 6 Department of Neurology, National Taiwan University Hospital, Taipei, Taiwan

**Keywords:** Interventional cardiology, Outcomes research

## Abstract

The safety of endovascular revascularization in patients with carotid artery near occlusion (CANO) is unknown. We aimed to evaluate the peri-procedural risk in CANO patients receiving carotid artery stenting (CAS). A prospective data base with retrospective review was performed to identify patients who underwent CAS with CANO from July 2006 to July 2020, and had at least 1-month clinical follow-up data. The primary endpoints were stroke, hyperperfusion syndrome, and death within 30 days after CAS. A total of 198 patients with carotid artery stenosis were enrolled including 92 patients with CANO and 106 age and sex-matched patients with 70–99% conventional carotid stenosis. Full distal carotid collapse was found in 45 CANO patients (45/92, 49%). The technical success rate was 100%. The CANO patients had significantly longer lesion lengths compared with those of the non-CANO group. The incidence of hyperperfusion syndrome was comparable (CANO: 2.2%, non-CANO: 0.9%, P = 0.598). The risks of ischemic stroke and death within 30 days were 1.1% and 0% in the CANO group; and 1.9% and 0.9%, in the non-CANO group, respectively, without statistical difference. In conclusion, CAS is safe for patients with CANO, with a similar low 30-day peri-procedural event rate comparable to those of non-CANO.

## Introduction

**Figure 1:**
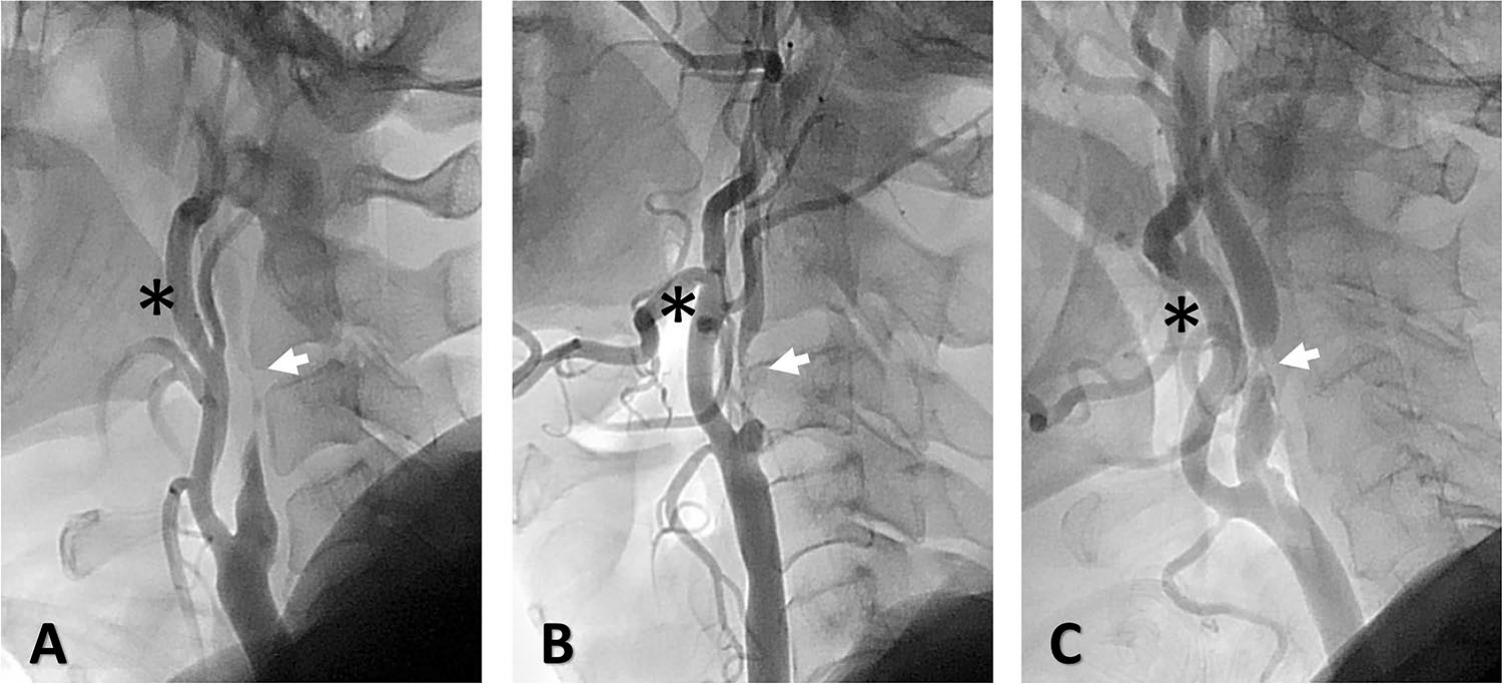
Illustration of different variants of carotid stenosis. (**A**) Carotid artery near occlusion with full collapse (**B**) Carotid artery near occlusion without full collapse (**C**) Severe conventional carotid stenosis without near occlusion. Black star: external carotid artery. White arrow: internal carotid artery.

Carotid artery near occlusion (CANO) is defined as a reduced internal carotid artery (ICA) lumen diameter distal to a prominent bulb stenosis^1^. The actual stroke risk in CANO remains elusive. There are 2 subtypes of CANO, severe form with full collapse (thread like distal ICA lumen) versus partial form without full collapse (a normal-appearing but narrowed distal ICA lumen) (Fig. 1), and their prognosis could be different^2^. Current guideline suggests medical treatment for CANO^3^, but the recommendations are based on experts’ consensus on an assumed low stroke risk and high revascularization complication rates. Recent studies, however, suggested high stroke risk and poor clinical outcome in CANO with full collapse, compared with those of severe carotid stenosis without CANO features^4,5^. In addition, recent meta-analysis showed that medical treatment alone may not be enough^6^. Evidences are urgently needed for the optimal management recommendation in CANO patients^4,7^.

None of the modern prospective carotid revascularization trials, such as the Carotid Revascularization Endarterectomy versus Stenting Trial (CREST)^8,9^ and International Carotid Stenting Study (ICSS)^10,11^, included CANO patients. Several recent observational studies reported improvement in cerebral hemodynamics and cognitive functions, and reduced risk of stroke after carotid artery stenting (CAS) in CANO patients^12–15^. However, the technical difficulty and risk of periprocedural complications, especially hyperperfusion syndrome, may be underestimated and should be investigated^12,16^. We hereafter reported our results of CAS in CANO patients.

## Methods

### Patients

Patients underwent CAS for carotid stenosis at the National Taiwan University Hospital from July 2006 to July 2020, with complete 1-month clinical follow-up, were reviewed from a prospective CAS database. Of these patients, 92 CANO patients were identified, and another 106 age- and sex-matched patients with conventional internal carotid stenosis (70–99% diameter narrowing without CANO features) were enrolled as controls. The definition of CANO was based on the previously reported angiographic criteria as evidences of narrowing of the post-stenotic ICA. The ipsilateral ICA/ contralateral ICA ratio, ipsilateral ICA/ipsilateral external carotid artery (ECA) ratio, the evidence of reduced flow in the distal ICA including delayed arrival of contrast into the distal ICA or evidence of intracranial collateral flow toward the affected cerebral hemisphere from other arterial territories were used to help correctly define post-stenotic distal ICA narrowing^1,17^. Patients with post-stenotic distal ICA narrowing due to anatomic variants such as ICA asymmetry associated with circle of Willis variations were excluded from this study^18^. CANO with full collapse was defined as a distal ICA lumen diameter ≤ 2 mm and/or ipsilateral ICA/contralateral ICA diameter ratio ≤ 0.42^2^. The angiograms were retrospectively reviewed by 2 independent investigators experience in cerebral angiography, who were blinded to the procedural and clinical information. In case of disagreement, consensus was reached after joint review and discussion.

The patients’ medical information, including demographics, medications, and neurological conditions were reviewed. A “symptomatic” lesion was defined as causing hemispheric transient ischemic attack (TIA), transient monocular blindness, or a stroke within 6 months, associated with ipsilateral carotid artery stenosis according to the North American Symptomatic Carotid Endarterectomy Trial (NASCET) definitions^19^. All patients received baseline biochemistry studies, non-invasive image studies including carotid duplex, computed tomography (CT) angiography or magnetic resonance (MR) angiography, before conventional angiography and ICA stenting. All patients were followed at the outpatient clinic after 7 days and 30 days post discharge, with full neurological evaluation. This study was approved by the Institutional Review Board of National Taiwan University Hospital and was performed in accordance with relevant guidelines and regulations. Informed consent was obtained from all patients before enrollment.

### Intervention protocol

All patients received 7 days of standard dual antiplatelet therapy before the procedure. All interventions were performed via an 8Fr femoral sheath, with heparin administered to maintain an activated clotting time within 200–250 s. The severity of ICA stenosis was measured according to NASCET method^19^. Intracranial collaterals from ipsilateral and contralateral origins were carefully examined. The target common carotid artery (CCA) was engaged with an 8Fr JR 4 guiding catheter. A distal embolic protection device (EPD) was deployed if technically feasible. After careful wiring and balloon pre-dilatation if necessary, properly sized self-expanding stents were deployed to scaffold the stenosis. Balloon post-dilation was done to achieve adequate stent expansion. Technical success was defined as residual diameter stenosis ≤ 20% and establishing grade 3 Thrombolysis in Cerebral Infarction (TICI) antegrade flow. After the procedure, all patients were sent to the intensive care unit for continuous blood pressure and neurological symptoms monitoring for 24 h. During the procedure and the intensive care unit stay, the systolic blood pressure was strictly controlled within 100 to 140 mmHg. All patients received 100 mg aspirin and 75 mg clopidogrel daily for at least 1 month after the intervention.

### Outcome definitions

Hyperperfusion syndrome, ischemic stroke, and mortality within 30 days after CAS were defined as the primary endpoints. Hyperperfusion syndrome was defined as ipsilateral headache, seizures, focal neurological deficits, encephalopathy, intracranial hemorrhage with evidence of hyperperfusion (on transcranial Doppler, single photon emission tomography, CT or MR perfusion imaging) within 30 days after CAS, without evidence of new cerebral ischemia, carotid stent occlusion, or metabolic causes^20^. Ischemic stroke was defined as an acute neurologic event with focal symptoms and signs, lasting for ≥ 24 h, with focal cerebral ischemia^8^. Ischemic stroke was categorized as being a major stroke if the National Institute of Health Stroke Scale (NIHSS) score was ≥ 9, or it was fetal or disabling^8^. TIA was defined as an episode of focal neurologic dysfunction attributed to focal cerebral ischemia, with resolution within 24 h.

### Statistical analysis

Statistical analysis was performed using SPSS version 25 for Windows (SPSS Inc., IL, USA). The data were first tested for normality using Kolmogorov–Smirnov test. Normal distribution data were expressed as mean ± standard deviation. Categorical data were expressed as number and percentage (%). Differences between proportions were calculated using the chi-square test or Fisher’s exact test. Comparisons of data between two groups were performed using the independent T test (normally distributed data). A two-sided P value less than 0.05 was defined as being statistically significant.

## Results

### Patients demographics and angiographic characteristics

A total of 92 patients with CANO and 106 patients with conventional carotid stenosis (NASCET diameter stenosis 70–99%) were enrolled in this study. Of the patients with CANO, 45 patients (49%) had full collapse. The demographic and angiographic characteristics are listed in Table 1. Less patients in the CANO group had history of head/neck radiation, but more co-morbid coronary artery disease. Within the CANO group, more patients with full collapse were symptomatic than those without. The delay from the last presenting events of neurological symptoms to CAS in the symptomatic patients were 67.4 ± 44.9 days in the CANO group and 75.9 ± 45.0 days in the non-CANO group (P = 0.469). The lesion lengths were significantly longer in the CANO group, especially in those with full collapse group than in the without full collapse group.

**Table 1:** Demographics and angiographic data.

	CANO (N = 92)	Conventional stenosis (N = 106)	P value	CANO with full collapse (N = 45)	CANO without full collapse (N = 47)	P value¶
**Demographics**						
Age, year	70.5 ± 8.9	70.6 ± 9.3	0.965	70.0 ± 8.7	71.0 ± 9.1	0.584
Male sex	76 (83%)	85 (80%)	0.663	37 (82%)	39 (83%)	0.924
BMI	24.7 ± 3.7	24.1 ± 3.5	0.255	24.4 ± 3.4	25.0 ± 3.9	0.434
Symptomatic disease*	43 (47%)	38 (36%)	0.120	27 (60%)	16 (34%)	0.013
Smoking	57 (62%)	55 (52%)	0.154	28 (62%)	29 (62%)	0.959
CAD	71 (77%)	66 (62%)	0.023	37 (82%)	34 (72%)	0.259
DM	36 (39%)	36 (34%)	0.451	21 (47%)	15 (32%)	0.147
HTN	74 (80%)	85 (80%)	0.965	35 (78%)	39 (83%)	0.530
Dyslipidemia	76 (83%)	80 (75%)	0.220	39 (87%)	37 (79%)	0.315
Hx of H&N RT	12 (13%)	34 (32%)	0.002	3 (7%)	9 (19%)	0.076
Atrial fibrillation	7 (8%)	8 (8%)	0.987	4 (9%)	3 (6%)	0.711
**Angiographic characteristics**						
Target-lesion length, mm	17.4 ± 8.6	14.4 ± 3.7	0.002	19.7 ± 10.9	15.2 ± 4.7	0.014
Ipsilateral CCA lesion	9 (10%)	15 (14%)	0.348	3 (7%)	6 (13%)	0.325
Contralateral ICA total occlusion	8 (9%)	14 (13%)	0.314	5 (11%)	3 (6%)	0.421
Ipsilateral ECA total occlusion	5 (5%)	0 (0%)	0.020	2 (4%)	3 (6%)	1.000
Criteria of CANO						
ICA < contralateral ICA^#^	84 (100%)	0 (0%)	< 0.001	40 (100%)	44 (100%)	NA
ICA diameter < ipsilateral ECA^#^	54 (62%)	0 (0%)	< 0.001	38 (88%)	16 (36%)	< 0.001
Delayed flow of ICA	53 (58%)	6 (6%)	< 0.001	34 (76%)	19 (40%)	0.001
Intracranial collateral	64 (70%)	15 (14%)	< 0.001	42 (93%)	22 (47%)	< 0.001
Ipsilateral ICA diameter, mm	2.5 ± 1.5	5.7 ± 0.7	< 0.001	1.2 ± 0.7	3.7 ± 0.8	< 0.001
Ipsilateral ICA/contralateral ICA ratio^#^	0.44 ± 0.26	1.10 ± 0.53	< 0.001	0.21 ± 0.13	0.66 ± 0.14	< 0.001
Ipsilateral ICA/ipsilateral ECA ratio^#^	0.76 ± 0.68	1.67 ± 0.54	< 0.001	0.44 ± 0.78	1.01 ± 0.36	< 0.001

BMI: body mass index; CAD: coronary artery disease; DM: diabetes mellitus; HTN: hypertension; Hx: history; CVA: cerebral vascular accident; H&N RT: head and neck radial therapy; CCA: common carotid artery; TICI: the thrombolysis in cerebral infarction; ICA: internal carotid artery; ECA: external carotid artery.

*Symptomatic disease: a hemispheric transient ischemic attack (distinct focal neurologic dysfunction) or monocular blindness persisting less than 24 h or a stroke with persistence of symptoms or signs for more than 24 h within the previous 120 days before the index procedure.

^#^Cases with contralateral ICA total occlusion or ipsilateral ECA total occlusion were excluded in the related analysis.

^¶^P value of comparing CANO patients with and without full collapse.

### Procedural data

The procedural data are shown in Table 2. The technical success rate was 100%, and no device failure or procedural strokes were observed. An EPD was used in all except for two CANO patients. Of note is that some of the CANO lesions, especially those with full collapse, even required pre-dilatation before EPD deployment. CANO lesions were also more likely to receive pre-dilatation to facilitate stent delivery due to tight stenosis. But direct stenting without pre-dilatation was the majority in the current cohort. Three (3.3%) patient in CANO group and two (1.9%) patients in the conventional stenosis group experienced TIA, but all recovered within 12 h after CAS without neurological deficit.

**Table 2:** Procedural data and events.

	CANO (N = 92)	Conventional stenosis (N = 106)	P value	CANO with full collapse (N = 45)	CANO without full collapse (N = 47)	P value^¶^
Pre-dilatation before EPD	8 (9%)	2 (2%)	0.029	8 (18%)	0 (0%)	0.002
EPD usage	90 (98%)	106 (100%)	0.127	43 (96%)	47 (100%)	0.144
Pre-dilatation before stent deployment	28 (30%)	12 (11%)	0.001	17 (38%)	11 (23%)	0.134
Stent across carotid bifurcation	80 (87%)	92 (87%)	0.973	39 (87%)	41 (87%)	0.936
Post-dilatation	89 (97%)	96 (91%)	0.080	43 (96%)	46 (98%)	0.532
Bradycardia*	13 (14%)	12 (11%)	0.553	5 (11%)	8 (17%)	0.416
Hypotension*	27 (29%)	20 (19%)	0.084	16 (36%)	11 (23%)	0.201
TIA	3 (3%)	2 (2%)	0.665	2 (4%)	1 (2%)	0.613

EPD: distal Embolic protection device; TIA: transient ischemic attack.

*Bradycardia and hypotension defined by the needs of chronotropic/vasoactive agents use after stenting.

^¶^P value of comparing CANO patients with and without full collapse.

### Endpoints

The clinical outcomes are listed in Table 3. Hyperperfusion syndrome occurred in two patients (2.2%) in the CANO group: one patient presented with headache, focal weakness, and aphasia 1 day after CAS. Perfusion CT showed reginal brain swelling and hyperperfusion. Another patient experienced headache and transient right upper limb weakness, resolved 12 h after the CAS. The CT showed mild left brain swelling and the hyperperfusion syndrome was suspected. Hyperperfusion syndrome occurred in one patient (0.9%) in the non-CANO group who presented with acute onset headache and focal seizure 1 week after CAS. Magnetic resonance imaging documented regional cerebral hyperperfusion. All these patients recovered completely after conservative treatment, and new infarction was ruled out by follow-up imaging studies.

**Table 3:** Clinical outcomes.

	CANO (N = 92)	Conventional stenosis (N = 106)	P value	CANO with full collapse (N = 45)	CANO without full collapse (N = 47)	P value^¶^
Hyperperfusion syndrome	2 (2.2%)	1 (0.9%)	0.598	0 (0.0%)	2 (4.3%)	0.495
Ischemic stroke	1 (1.1%)	2 (1.9%)	1.000	1 (2.2%)	0 (0.0%)	0.489
Major ipsilateral	1 (1.1%)	1 (0.9%)	1.000	1 (2.2%)	0 (0.0%)	0.489
Major non-ipsilateral	0 (0.0%)	0 (0.0%)	NA	0 (0.0%)	0 (0.0%)	NA
Minor ipsilateral	0 (0.0%)	1 (0.9%)	1.000	0 (0.0%)	0 (0.0%)	NA
Minor non-ipsilateral	0 (0.0%)	0 (0.0%)	NA	0 (0.0%)	0 (0.0%)	NA
Mortality	0 (0.0%)	1 (0.9%)	1.000	0 (0.0%)	0 (0.0%)	NA
All stroke and mortality	1 (1.1%)	2 (1.9%)	1.000	1 (2.2%)	0 (0.0%)	0.489

Ischemic stroke was categorized as major stroke if the National Institute of Health Stroke Scale (NIHSS) score was ≥ 9, or as a fetal or disabling stroke.

^¶^P value of comparing CANO patients with and without full collapse.

Three ipsilateral strokes occurred within 30 days post CAS, including one (1.1%) major stroke in the CANO group, one major stoke (0.9%) and one minor stroke (0.9%) in the non-CANO group. The composite outcome of all stroke and mortality within 30 days after CAS was 1.1% in the CANO group and 1.9% in the non-CANO group.

## Discussion

The prevalence of CANO was variable in the past literatures, due to different diagnostic methods, criteria, and the study designs. Recent systemic review suggested that the prevalence of CANO in symptomatic carotid stenosis may be as high as 34%^21^. CANO with full collapse can be easily misdiagnosed as complete occlusion, and CANO without full collapse may simply be classified as conventional stenosis. Therefore, the prevalence of CANO may be under-reported in clinical routine practice^22^, and careful examination of diagnostic images using a uniform definition is important.

The severity of carotid stenosis is known to be generally associated with the risk of ipsilateral ischemic stroke in both symptomatic and asymptomatic patients^19,23–26^. As the stenosis progresses to the near-occlusion, however, the reported risk becomes controversial. Rothwell et al. reported a relatively low risk of ipsilateral stroke in medically treated patients with severe carotid stenosis with post-stenotic narrowing of the ICA^27^. In contrast, Paciaroni et al. reported that 30% of medically treated severe carotid stenosis would progress to total occlusion, and that this was associated with impaired brain perfusion and ipsilateral stroke in the long-term follow-up^28^. In a recent study, Gu et al. reported that symptomatic CANO was associated with a high short-term risk of recurrent ipsilateral ischemic stroke, especially those with distal vessel full collapse^29^. The natural history of CANO, therefore, remains elusive.

Current guideline recommends conservative treatment for patients with CANO, unless it is associated with recurrent ipsilateral symptoms despite optimal medical therapy (OMT)^3^. Recent prospective carotid revascularization trials such as CREST and ICSS, unfortunately, have excluded these patients^8–11^. Therefore, the current guideline recommendations are based on previous surgical endarterectomy trials data and do not consider contemporary advance in surgical techniques, OMT, or carotid stenting. A recent meta-analysis suggested the risk of stroke was higher in CANO patients receiving OMT alone, compared with those receiving revascularization (OMT 8.4%, endarterectomy 1.5%, CAS 1.8%)^30^. Modification of management guidelines for CANO is therefore necessary, mandating studies to collect more data.

Perioperative stroke rate was 6.3% in CANO patients receiving endarterectomy in the NASCET trial, similar to that in patients with 70–94% stenosis^23^. Kim et al. reported similar periprocedural complication rates in symptomatic CANO patients receiving revascularization, 5.9% in CAS and 3.5% in endarterectomy^13^. In our study, the 30-day ischemic stroke and death rate in the CANO patients was only 1.1%, which is significantly lower than in prior reports. In addition, the 30-day complication rate was similar in the CANO and non-CANO patients. Overall improvement of CAS devices and techniques, high percentage of embolic protection device usage, and direct stent delivery without balloon pre-dilatation were the possible explanations.

Hyperperfusion syndrome is a potentially life-threatening complication following carotid revascularization, especially in those with critical carotid stenosis. However, its risk has been under-reported and underestimated. In a recent meta-analysis, hyperperfusion was seen in up to 4.6% CAS procedures, and nearly half of these progressed to stroke^16^. Its incidence has rarely been described in previous studies in CANO^31,32^, which may be theoretically high due to the impaired hemodynamics and autoregulation. The incidence of hyperperfusion in this study, however, was low and comparable in patients with or without CANO. Possible explanations included the modest procedural anticoagulation, strict blood pressure control protocol, and high incidence of established intracranial collaterals. Recent study has identified insufficient collateral and unregulated hypertension were predictors of hyperperfusion after endarterectomy^33^. Admittedly, we might have missed hyperperfusion with only subtle non-specific symptoms. It should be emphasized that awareness and proper management of hyperperfusion in CANO revascularization is very important, and modalities such as transcranial Doppler may be mandatory to improve detection and monitoring in future research.

## Limitation

There were several limitations to this study. First, this was a retrospective study with a limited number of cases. Second, we only provided 30-day event rates to examine the safety of CAS in CANO and non-CANO patients. Longer-term effect of CAS in CANO, therefore, cannot be demonstrated. Third, the delay from the last presenting events of neurological symptoms to CAS in the symptomatic patients were relatively long. Therefore, the findings of this study might not be applicable in urgent CAS. Finally, we only investigated hyperperfusion with clinical suspicion, so its incidence might be underestimated.

## Future considerations and clinical implications

Endovascular revascularization is an alternative for carotid artery stenosis in patients with high surgical risk. Current guideline suggests OMT for CANO patients, and the benefit and safety of CAS in CANO remain unclear despite encouraging recent data. The present study compared results of CAS in severe carotid stenosis with and without CANO and demonstrated low 30-day peri-procedural event rates in CANO, comparable with those in non-CANO. Future large prospective trial including patients receiving OMT, endarterectomy, and CAS are mandatory to establish new management guidelines for CANO patients.

## Conclusion

The present study demonstrated low peri-procedural event rates of CAS in CANO patients, comparable to those in non-CANO patients.

## Abbreviations

CANO: Carotid artery near occlusion
CCA: Common carotid artery
CT: Computed tomography
ECA: External carotid artery
EPD: Embolic protection device
ICA: Internal carotid artery
MR: Magnetic resonance
NASCET: North American Symptomatic Carotid Endarterectomy Trial
NIHSS: National Institute of Health Stroke Scale
OMT: Optimal medical therapy
TIA: Transient ischemic attack
TICI: Thrombolysis In Cerebral Infarction
